# Electrolysis for the Treatment of Peridental Inflammations

**Published:** 1905-01

**Authors:** A. H. Macpherson

**Affiliations:** Philadelphia


### ELECTROLYSIS FOR THE TREATMENT OF PERIDENTAL INFLAMMATIONS.[1]

BY DR. A. H. MACPHERSON, PHILADELPHIA.

Electrolysis comes to our profession as a new treatment for inflammations arising in the peridental membrane. It simply does its own work, with no tendency to crowd out other curative agents. Immediate relief may be given in all stages of inflammation; if little or no pus has formed, electrolysis will hasten the formation and draw it to the surface. Where there is much swelling and accumulation of pus, electrolysis will cause the pus to discharge where the electrode is placed; the swelling will disappear, the muscles relax, the flow of blood will be increased, and pain will

[1] Read before the Academy of Stomatology, Philadelphia.

cease. Where there is intense suffering from pyorrhœa the treatment will give immediate relief and remove any accumulation of pus.

Electrolysis is the dissolution of a chemic compound by an electric current. The current passing from the positive pole to the negative pole passes through resistant tissue, stimulating the flow of blood, or foreign matter, that may exist in its path. The blood-vessels keep the blood from coming to the surface while pus, which is found in a free state, will flow where the electric current may direct. The direction of the current is toward the negative pole and nothing can prevent its progress. Pus that may exist in the body will be found and brought to the surface where the negative electrode is placed.

Electrolysis is master of the situation, and its work is thorough and complete, marvellous as it may seem. This wonderful power we are permitted to control, and by it relieve days of suffering. The operation is from fifteen to twenty minutes duration and almost painless. Electrolysis used in dental practice stimulates the flow of blood, and the patient realizes the good effects through the entire system.

Electrolysis may only be used from a series of batteries giving a moderate amount of voltage without a series current controller. The amount of current is obtained by a cell selector, or the electric light current may be passed through a genuine shunt controller. The positive pole is applied with a sponge at the nerve-centres at the back of the head, or preferably held in the hand, any rings worn being first removed; the negative electrode is applied where it is desired to remove pus.

One hundred volts will destroy tissue, while it only requires from five to twenty volts for this work. Electrolysis has been used by the medical profession for the past twenty-five years for the removal of tumors, superfluous growths, and birth-marks. This method was discovered by Mr. F. Geiger, an electrician of Philadelphia, who has had many years of experience in medical electricity. For fifteen years Mr. Geiger has applied electrolysis to relieve abscessed conditions of his teeth, and always found relief from untold suffering.

The polarity should always be tested where the electric light current is used. Turn on all the current and place the poles in a glass of water; the negative pole will produce bubbles. Should the positive pole be placed over the abscess, the pus will be scattered and drain into the system.

What proof have we that it is pus? may be asked. By the relief given, by the quick healing of an abscess, and by chemical analysis made by Dr. I. N. Broomell, who witnessed an application of electrolysis at the Pennsylvania State meeting, last summer, at which time I gave a demonstration. The patient then treated had been suffering severe pain in an inferior cuspid which was badly decayed. The doctor—my patient—said, "If the pain does not stop in the next two hours I shall have the tooth extracted." The tooth had been aching for twenty-four hours, with no sleep for the sufferer. The question presented itself, Has pus formed? A member of the society standing near suggested that no doubt there was some formation of pus. There was no swelling and little or no soreness about the tooth. I applied electrolysis, and in less than five minutes there was pus discharging at the negative electrode. The application was continued for about twenty minutes, twenty-four volts being used. More than a spoonful of pus was removed and the pain relieved. Mr. Geiger claims great results yet to be attained by electrolysis. It is an easy and simple operation for him to cure a boil on the neck, and he has done it scores of times. What is more wonderful, he believes it possible to cure blood-poisoning and lockjaw, and would gladly put his belief to a test.

Gentlemen, there is a thought to which I must give voice, and that is, what a blessing to humanity it would be if it were possible for Mr. Geiger to give all of his time and attention to this work! As it is he has very little time to devote to this branch. I believe his heart is in this work, and that he would gladly give himself to study and research along these lines. I have found electrolysis to be a friend in need many times. Its influence is for good, as it stimulates the dentist to more exacting and thorough work and greater faith and confidence in his efforts to become a master of his profession.

